# Global DNA methylation profiling reveals new insights into epigenetically deregulated protein coding and long noncoding RNAs in CLL

**DOI:** 10.1186/s13148-016-0274-6

**Published:** 2016-10-12

**Authors:** Santhilal Subhash, Per-Ola Andersson, Subazini Thankaswamy Kosalai, Chandrasekhar Kanduri, Meena Kanduri

**Affiliations:** 1 Department of Medical Genetics, Institute of Biomedicine, Sahlgrenska Academy, Gothenburg University, Gothenburg, Sweden; 2 Department of Internal Medicine and Clinical Nutrition, Institute of Medicine Sahlgrenska Academy, Gothenburg University, Gothenburg, Sweden; 3 Department of Internal Medicine, Södra Älvsborg Hospital, Borås, Sweden; 4 Department of Clinical Chemistry and Transfusion Medicine, Institute of Biomedicine, Sahlgrenska Academy, Gothenburg University, S-413 45 Gothenburg, Sweden

**Keywords:** DNA methylation, Chronic lymphocytic leukemia, Hyper/hypomethylated regions, Repetitive elements and noncoding RNAs

## Abstract

**Background:**

Methyl-CpG-binding domain protein enriched genome-wide sequencing (MBD-Seq) is a robust and powerful method for analyzing methylated CpG-rich regions with complete genome-wide coverage. In chronic lymphocytic leukemia (CLL), the role of CpG methylated regions associated with transcribed long noncoding RNAs (lncRNA) and repetitive genomic elements are poorly understood. Based on MBD-Seq, we characterized the global methylation profile of high CpG-rich regions in different CLL prognostic subgroups based on IGHV mutational status.

**Results:**

Our study identified 5800 hypermethylated and 12,570 hypomethylated CLL-specific differentially methylated genes (cllDMGs) compared to normal controls. From cllDMGs, 40 % of hypermethylated and 60 % of hypomethylated genes were mapped to noncoding RNAs. In addition, we found that the major repetitive elements such as short interspersed elements (SINE) and long interspersed elements (LINE) have a high percentage of cllDMRs (differentially methylated regions) in IGHV subgroups compared to normal controls. Finally, two novel lncRNAs (hypermethylated *CRNDE* and hypomethylated *AC012065.7*) were validated in an independent CLL sample cohort (48 samples) compared with 6 normal sorted B cell samples using quantitative pyrosequencing analysis. The methylation levels showed an inverse correlation to gene expression levels analyzed by real-time quantitative PCR. Notably, survival analysis revealed that hypermethylation of *CRNDE* and hypomethylation of *AC012065.7* correlated with an inferior outcome.

**Conclusions:**

Thus, our comprehensive methylation analysis by MBD-Seq provided novel hyper and hypomethylated long noncoding RNAs, repetitive elements, along with protein coding genes as potential epigenetic-based CLL-signature genes involved in disease pathogenesis and prognosis.

**Electronic supplementary material:**

The online version of this article (doi:10.1186/s13148-016-0274-6) contains supplementary material, which is available to authorized users.

## Background

High-throughput next-generation sequencing techniques, with single base pair resolution have become increasingly feasible, along with the existing genomic and transcriptome sequencing methodologies. These techniques have been successfully used to understand the functional role of DNA methylation in leukemia development and progression, including CLL. Somatic hypermutations of the IGHV gene have been shown to be a strong prognostic marker in CLL, where CLL patients with an unmutated IGHV gene have poor prognosis and shorter survival time compared to IGHV-mutated CLL patients [1, 2]. Previously, using high-resolution 27K/450K methylation arrays in CLL, we analyzed the global methylation profiles of well-characterized prognostic groups such as IGHV-mutated and IGHV-unmutated CLL subsets [3–6]. Our data identified a large number of differentially methylated genes with prognostic implications for the CLL prognostic subgroups, and importantly, we found that the methylation patterns were stable over time and between the compartments [3, 5]. In addition, using 450K methylation arrays and whole genome bisulphite sequencing (WGBS) techniques, a recent investigation characterized the DNA methylomes of CLL patients and found that differential methylation in the gene body may have functional and clinical implications in leukemogenesis [7, 8]. The most common methodologies used in all these CLL studies include microarray and sequencing methods which are based on bisulfite conversion of genomic DNA for differentiating 5-methyl cytosine (5mC) from cytosine (C).

However, bisulfite conversion-based methodologies have some drawbacks; these methods fail to differentiate between 5mC and other epigenetic modifications such as 5hmC (hydroxyl methyl cytosine) and 5cmC (carboxyl methyl cytosine) [9, 10], and they may also not be the best methods for characterizing repeat sequences in the genome. Instead, other techniques like affinity-based enrichment methods such as MBD-Seq or Methylated DNA immunoprecipitation, followed by sequencing (MeDIP-seq) can overcome these drawbacks and provide genome-wide coverage of CpG methylation in a PCR-unbiased manner. These immunoprecipitation-based enrichment of CpG methylated DNA methods in CLL provide DNA methylation profiling for both protein coding and noncoding RNAs, as well as repeat regions which have not yet been studied. A recent study showed very good correlation between 450K methylation array and MeDIP-seq on a genome-wide scale. However, MeDIP-Seq allowed wider interrogation of methylated regions in the human genome, including some nonreference sequences that were not included in the array and also the methylation of repetitive elements [11].

Noncoding RNAs (ncRNAs) have been shown to regulate important biological functions such as maintenance of nuclear architecture, X-chromosome inactivation [12], and genomic imprinting [12, 13]. ncRNAs can be broadly classified into long noncoding RNAs (lncRNAs), microRNAs (miRNAs), antisense RNAs, small nuclear RNAs (snRNAs), and small nucleolar RNAs (snoRNAs). Like proteins, ncRNA modulate transcription and play regulatory roles in controlling the localization and activity of proteins [14–17]. The precise distribution and temporal expression of ncRNAs in the genome are important for cellular homeostasis. Deregulation of the expression of ncRNAs leads to several disorders including cancer [16, 18, 19], and recent studies underline the emerging role of ncRNAs as biomarkers in different malignancies [20–22]. Even though global differential expression patterns of ncRNAs were observed between CLL cells and corresponding healthy controls [23, 24], studies on the novel epigenetically deregulated ncRNAs in CLL are limited.

In order to investigate CLL-associated differentially methylated genes compared to normal healthy controls, we performed MBD-Seq to ascertain the global distribution of the methylomes between five IGHV-mutated and five IGHV-unmutated CLL patient samples. Additionally, we also compared the methylomes of each subgroup with healthy age-matched controls, against PBMCs and sorted B cells separately. This is the first MBD-Seq-based CLL study, revealing many CLL-specific significantly methylated protein coding genes, noncoding RNAs, and certain repetitive regions with potential prognostic significance.

## Methods

### Patient samples, ethics, clinical data, cell lines, and cell culture conditions

In the present study, a total of 70 CLL patients (35 IGHV-unmutated samples + 35 IGHV-mutated samples) were included. All patients were diagnosed according to recently revised criteria [25] and the tumor samples were collected at the time of diagnosis. The patients in the study were included from different hematology departments in the western part of Sweden after written consent had been obtained. Only CLL peripheral blood mononuclear cells (PBMC) samples with a tumor percentage of leukemic cells ≥70 % were selected in this study. Clinical and molecular data are summarized in Additional file 1A and B. PBMCs from peripheral blood of age-matched normal healthy controls was prepared using the Ficoll extraction method and normal CD+19 positive sorted B cell DNA from eight healthy age-matched controls were bought from a company (3H Biomedicum, Uppsala, Sweden). Two CLL cell lines (HG3 [26] and MEC1 [27]) and one Burkitt lymphoma B cell line (RAMOS) [28] were used for DAC treatment experiments. All cell lines were cultured in RPMI 1640 with glutamine (Invitrogen, Carlsbad, USA) supplemented with 10 % fetal bovine serum and 1× penicillin/streptomycin (FBS; Invitrogen, Carlsbad, USA).

### DNA and RNA extractions

DNA and RNA were extracted from CLL PBMC samples using DNA and RNA Extraction Kit (Qiagen, Hilden, Germany) according to the manufacturer’s protocol. For total cDNA preparation, reverse transcription (RT) was performed using Superscript III FS synthesis supermix kit (Life technologies, Carlsbad, USA) according to the manufacturer’s protocol.

### Methyl-binding domain sequencing and data preparation

Purified genomic DNA from CLL patient samples were subjected to sonication using bioruptor (Diagenode, Liege, Belgium) to generate fragment sizes of around 100 to 350 bp. The fragmented DNA was then subjected to MethylMiner^TM^ methylated DNA kit enrichment according to the manufacturer’s protocol and the enriched methylated DNA was purified using single fraction extraction with buffer containing 2000 mM NaCl. The eluted DNA was purified and sent for downstream library construction and high-throughput MBD-Seq using the Illumina HIseq2000 platform. The analysis has been done using five IGHV region-mutated and five IGHV region-unmutated patients samples along with normal PBMC and normal B cell as control samples. The raw sequenced reads (FASTQ files) from Illumina for two sorted BCELL, two PBMC, five IGHV-mutated, and five IGHV-unmutated samples are deposited on European Nucleotide Archive (ENA) under project ID “PRJEB12693” and can be accessed via following link http://www.ebi.ac.uk/ena/data/view/PRJEB12693. The raw reads from sequencing were cleaned for adaptors using Trimmomatic [28], and bioinformatics analysis has been performed on those clean reads. The hg19/GRCh37 genome version was used to map obtained 49-bp cleaned reads. The alignment was performed using Bowtie v1.0 aligner by allowing up to two mismatches. It is a short-read aligner supports up to length of 50 bp [29]. We used an additional parameter *-m 6* in Bowtie to reduce the number of multiple aligned reads.

### Differential methylation and functional significance (association of differentially methylated regions to genes in the genome)

**Fig. 1:**
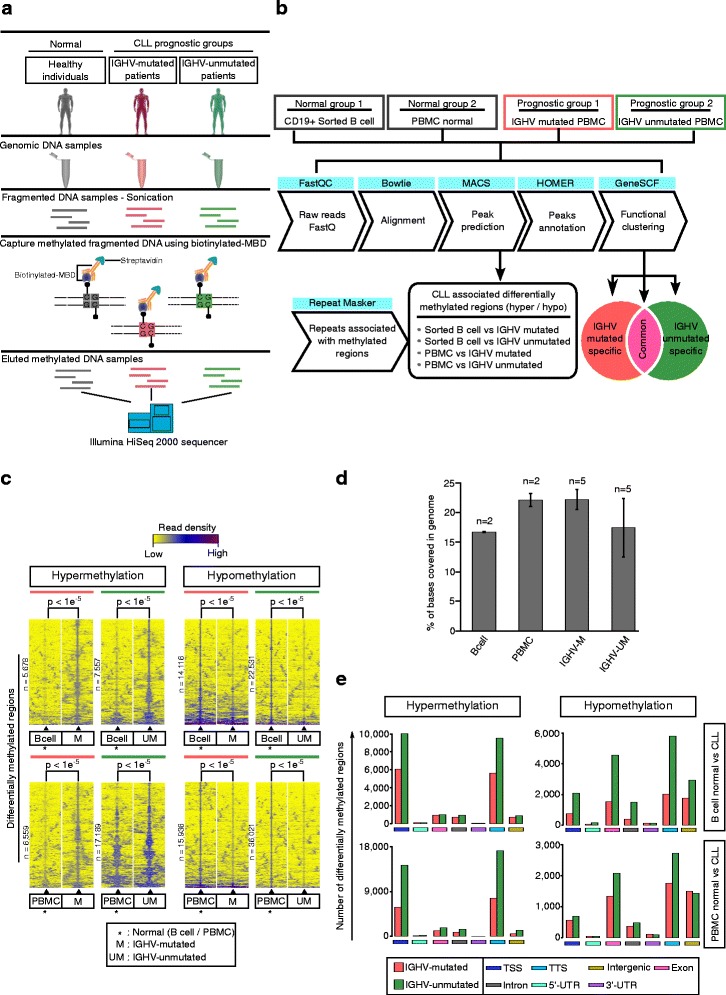
The global methylation levels and identification of differentially methylated regions (DMRs) in CLL patient samples. **a**, **b** Analysis pipeline used to find CLL-associated differentially methylated regions (DMRs). **c** Differentially methylated regions (DMRs, hypermethylated and hypomethylated) in IGHV-mutated and IGHV-unmutated samples over sorted B cells and normal PBMC. The enrichments shown in the heatmap were within a ±3 kb window from differentially methylated region (DMRs). **d** The *bar graph* shows the overall percentage of genome covered by in normal and prognostic CLL groups. **e** The *bar graphs* in (**e**) show the difference in distribution of hypermethylated and hypomethylated patterns across the genome. The peaks used for assigning the genomic regions were derived from MACS with a significance of *p* < 1E−05

The differentially methylated regions were predicted using MACS v1.4.2 peak caller [30] by not considering duplicate reads at exact location. The analysis has been carried out by assigning four different groups, two prognostic groups (IGHV-mutated and IGHV-unmutated PBMC), and two normal group (sorted B cell and PBMC normal). The normal groups were used as control in MACS and obtained positive peaks (enriched in prognostic groups) were termed as hypermethylated over normal whereas the negative peaks as hypomethylated (enriched in normal sample). This analysis was done using each control groups as different comparison (B cell comparison and PBMC comparison). All obtained CLL cllDMRs presented in Fig. 1c were enriched with a *p* value < 1e−05 (represents peak score, 50) and the enrichment heatmaps were obtained from a standard R package. The global methylation levels (as shown in Fig. 1d) represents percentage of bases in the genome occupied by enriched regions in normal B cell, PBMC, and IGHV-mutated and IGHV-unmutated CLL samples, and the analysis was done using bedtools-genomecov.

### Association of cllDMRs with different genomic regions and analysis of repeat regions

**Fig. 2:**
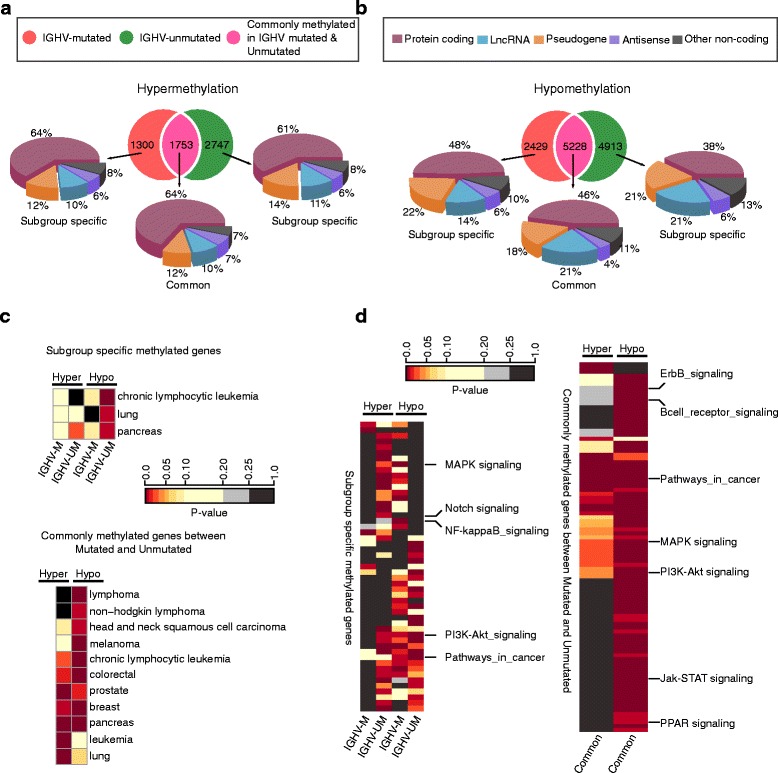
Association of DMRs to genes and the importance of associated genes (DMGs) in CLL over normal sorted B cell. **a**, **b** *Venn diagram* shows the overlap of differentially methylated genes (DMGs, hypermethylated and hypomethylated) between IGHV-mutated and IGHV-unmutated groups. The *pie chart* represents the percentage of different classes of genes such as protein coding, lncRNA, pseudogenes, antisense and other noncoding RNAs. **c** The heatmap shows enrichment of DMGs (*top*, subgroup specific and *bottom*, common DMGs) in different cancer types from Network of Cancer Genes (NCG 4.0). The cancer types are assigned and ranked using GeneSCF. The presented enrichment was filtered using a *p* value <0.01 with at least 5 % of total cancer genes covered by DMGs. **d** The heatmap shows the KEGG pathways obtained using DMGs from IGHV-mutated and IGHV-unmutated prognostic groups. The pathways were assigned and ranked using GeneSCF. The presented pathways are filtered using a *p* value <0.01 with at least 5 % of total pathway genes covered by DMGs (see the “Methods” section). The *left side of the heatmap* represents the subgroup specific (IGHV-mutated and IGHV-unmutated) hyper- and hypomethylated associated pathways; and the *right side of the heatmap* for common DMGs between IGHV-mutated and IGHV-unmutated groups (see the “Methods” section)

Association of cllDMRs to genes was done using HOMER [31] tool with Ensembl transcript annotation version GRCh37.74 and using default parameters in HOMER. We termed the genes associated with differentially methylated regions as differentially methylated genes (cllDMGs). After association, the genes from each prognostic subgroups (IGHV-mutated and IGHV-unmutated) were compared (for example, in Fig. 2a). There were some genes appeared in both subgroups, and we termed it as common cllDMGs. The genes which only fall in either of one subgroup we termed it as subgroup specific cllDMGs (IGHV-mutated specific and IGHV-unmutated specific cllDMGs). These terminologies were used for both hyper and hypomethylated genes (Fig. 2a, b) in all comparisons (B cell and PBMC normal comparisons). The percentage of repeat sequence covered by cllDMRs was obtained using RepeatMasker (http://repeatmasker.org) including all repeat elements as reference. We have used sequence from each peak region predicted by MACS to find sequence similarity (with minimum insertions or deletions) with known repeat elements using Repeatmasker. The clustering of repeat elements in Fig. 5a and Additional file 2: Figure S3A were done by “euclidean” as distance metric (complete-linkage clustering) using percentage of bases covered in cllDMRs by different repeat elements obtained.

### Pathway enrichment of cllDMGs and their enrichment in different cancer types

The pathway enrichment analysis and cancer enrichment analysis on cllDMGs was carried out with the help of a command line functional enrichment tool called GeneSCF v1.1 (Gene Set Clustering based on Functional annotation) [32, 33]. We used GeneSCF with parameters, two different database KEGG pathways and NCG (Network of cancer Genes 4.0) [34], Ensembl GRCh37.74 protein coding genes as background genes. The resulted KEGG pathways were filtered with a *p* value <0.05 with at least 5 % of total genes covered for particular pathway. For cancer enrichment analysis, we used a threshold of at least 5 % of total genes covered for corresponding cancer type. The GeneSCF ranks the pathways and cancer types with *p* values obtained from Fisher’s Exact test using total protein coding genes in the experiment as a background. The Fisher’s exact test is carried out based on overlaps between cllDMGs and the genes from corresponding databases (NCG or KEGG). The original list of all significant pathways (as presented in heatmaps from Fig. 2d, Additional file 2: Figure S1D and S2C) and cancer types (as presented in heatmaps from Fig. 2c, Additional file 2: Figure S1C and S2B) was listed as same order in Additional files 3 and 4.

### Processing and comparing cllDMGs with RNA sequencing expression data obtained from published CLL data set

Since the available processed dataset from Ferreira PG et al. [23] used different gene level annotation (GENCODE), we wanted to maintain the same annotation throughout our study (Ensembl). We obtained the raw data of RNA-seq samples for 96 patients (55 IGHV-mutated and 41 IGHV-unmutated prognostic groups) along with 9 normal B cell samples (Controlled Access ICGC dataset at the EGA, EGAD00001000258). The obtained samples are from paired-end with 76-bp length reads. The raw reads were subjected to adaptor cleaning using Trimmomatic, and the cleaned reads with 76-bp length was aligned to hg19 genome using a spliced read mapper Tophat v2.0.13 with default parameters. Reads were quantified for Ensembl annotation (GRCh37.74) using featureCounts from Subread packag v1.4.5 [35]. The obtained gene expression profile was normalized to reads per kilobase of transcript per million mapped reads (RPKM). The log-fold changes between B cell normal and two CLL groups (IGHV-mutated and IGHV-unmutated) were calculated based on obtained RPKM values. The statistics for differential expression between normal and CLL prognostic groups was obtained using Wilcoxon rank sum test in R package. The methylation patterns from pyrosequencing of CRNDE and AC012065.7 was inversely correlated with the gene expression patterns in RNA-seq dataset (Fig. 4a, b). The *p* values in this heatmap were presented as Wilcoxon rank sum test.

### Nearby protein coding genes analysis

The lncRNAs in cllDMGs from B cell and PBMC comparisons were used to extract nearby protein coding genes within 10 kb using bedtools “*--closest*.” Functional and cancer enrichment analysis for obtained nearby protein coding genes were performed by GeneSCF v1.1 using KEGG and NCG as reference database.

### Pyrosequencing and real-time quantitative PCR

Pyrosequencing was performed as previously described [36], using the Pyromark kit (Qiagen, Hilden, Germany) according to the manufacturer’s instructions. Pyrosequencing primers were designed using the PyroMark™ (Qiagen, Hilden, Germany) software (FP—5′GGAAAAGGGGAGGTAAAGAGG3′; RP—5′TACCTTTACAAAAATCCTACCAAAATA CTA3′; and sequencing primer—5′GGTAGTTTAGAAGTTTTTGTTAGTT3′ (280 bp product size) for *CRNDE*); (FP—5′AGTTTTTGTTTAGATTTTTGGTTGTTAGA3′; RP—5′AAAAA ATATATACAATTACACCAACTCAC3′; and sequencing primer—5′GTATTTTGTTGAATTA GAAGGA3′ (222-bp product size) for *AC012065.7*) and (FP—5′GTTTATAGATATGGTTAGA ATGGG3′; RP—5′TCCCCAATAACTAAAACTACAAACT3′; and sequencing primer—5′ATA TGGTTAGAATGGGT3′ (236-bp product size) for CLL IGHV-mutated specific SINE-ALU repeat). The analysis was performed using PyroMark™ Q24 advanced pyrosequencer instrument and the CpG site methylation percentage of target regions was calculated using the PyroMark Q24 advanced software. The expression levels of all genes were analyzed with Taqman gene expression assays (Applied Biosystems) (Hs00395639_m1 for *CRNDE*, custom assay designed primers for *AC012065.7* and Hs99999907_m1 for the b2-microglobin gene, which was used as an internal control). Differences in expression were calculated using the ∆∆Ct method.

### Overall survival analysis for validated genes

Correlations between overall survival and methylation or gene expression were calculated using the Kaplan-Meier method and the log-rank test. Differences were considered statistically significant when the *p* value was <0.05 (Fig. 4c, Additional file 2: Figure S3A).

## Results

### Experimental design and mapping of CLL-associated differentially methylated regions 

A brief overview of the work-flow used in this study is shown in Fig. 1a, b, which summarizes both the experimental work-flow and the computational pipeline used to analyze MBD-Seq data generated from CLL patients and normal healthy controls. In this study, the genomic DNA from five IGHV-mutated favorable prognostic and five IGHV-unmutated poor prognostic CLL samples were used, along with CD19+ sorted B cells and total PBMCs as normal controls obtained from two to three different pooled age-matched healthy controls (Fig. 1a). As an initial step of analysis, we extracted the differentially methylated regions, which are specifically hypermethylated or hypomethylated in CLL samples compared to the normal sorted B cell controls. These regions were named CLL-specific differentially methylated regions (cllDMRs) and were defined as enriched regions from IGHV-mutated and IGHV-unmutated samples compared to control samples with a *p* value <0.00001. Both cllDMRs and methylated repeat regions in the genome were fine mapped and then compared between different CLL prognostic subgroups and healthy controls as described in the Fig. 1b flow-chart. The obtained cllDMRs represent two groups, CLL-associated hypermethylated and hypomethylated regions across the genome (Fig. 1c). In Fig. 1c, all the differentially methylated regions with significant *p* values in comparison between CLL prognostic groups over two different normal controls, such as B cell comparison (upper panel) and PBMC comparisons (lower panel), are shown. The genes associated with cllDMRs were termed CLL-associated differentially methylated genes (cllDMGs).

### Characterization of cllDMRs across the genome

When compared the total percentage of genome covered by MBD-seq samples from CLL prognostic groups and normal healthy controls, we found that IGHV-unmutated samples showed less genome coverage compared to IGHV-mutated samples (Fig. 1d). The genome coverage denotes number of bases in the genome covered by aligned reads from corresponding samples. The overall genome coverage of IGHV-mutated samples were almost in the same range as normal controls such as PBMC and sorted B cells. The coverage levels of the normal PBMC sample were in a higher range compared to the normal sorted B cell sample as shown in the Fig. 1d. Interestingly, even though we found that the overall genome coverage of IGHV-mutated and IGHV-unmutated cllDMRs showed a similar pattern, there is a clear difference in distribution of cllDMRs between the hypermethylated and hypomethylated groups with respect to protein coding genes. According to the cllDMRs distribution data across the genome, hypermethylated cllDMRs are mostly enriched in promoter regions TSS (transcriptional start sites) and TTS (transcriptional termination sites), whereas hypomethylated cllDMRs are enriched in the gene body and intergenic regions (Fig. 1e).

The obtained cllDMRs were further associated with different classes of CLL-specific differentially methylated genes (cllDMGs), like protein coding and noncoding genes based on the overlapping genomic locations of cllDMRs as listed in Additional files 5 and 6. Nearly 50 % of cllDMRs, from IGHV-mutated and IGHV-unmutated CLL prognostic groups, map to ncRNAs (lncRNAs, microRNAs, snRNAs, snoRNAs, and pseudogenes) (Fig. 2a, b for B cell control and Additional file 2: Figure S1A and B for PBMC control comparisons). This data is in line with the published RNA-seq CLL study showing that many lncRNAs are differentially expressed in CLL compared to normal healthy controls and that DNA methylation could be one of main reasons behind their differential expression [23].

When enrichment for different cancer types was tested using hypermethylated and hypomethylated cllDMGs from CLL IGHV-mutated and IGHV-unmutated groups, the CLL cancer type was significantly enriched in both B cell and PBMC cllDMGs (Fig. 2c, B cell comparison; Additional file 2: Figure S1C, PBMC comparison; and Additional file 2: Figure S2B, common genes between B cell and PBMC comparison). In the B cell cllDMGs, along with CLL, lung and pancreas cancer types were also found to be significantly differentially methylated as shown in Fig. 2c. The detailed list of cancer type enrichments with the corresponding list of cllDMGs is shown in Additional files 3 and 4, and see the “Methods” section for enrichment analysis. On the other hand, hypermethylated and hypomethylated common cllDMGs (hypermethylated or hypomethylated in both prognostic subgroups) were highly enriched in several lymphomas, including CLL and other solid tumors like prostate, colorectal, and breast cancer. More importantly, the overlapped common cllDMGs (851 hypermethylated and 2061 hypomethylated) between B cell and PBMC control comparisons (Additional file 2: Figure S2A) showed significant enrichments in cancer types related to leukemia (Additional file 2: Figure S2B). These results show that most of the commonly deregulated cllDMGs are cancer associated genes, and they may have a functional role in CLL pathogenesis.

### Biological pathways deregulated by DNA methylation in CLL

We next performed a functional analysis to investigate pathways that were potentially deregulated by DNA methylation in CLL. Several novel as well as already implicated pathways in CLL have shown significant enrichments either in IGHV-mutated specific methylated (hypo or hyper) genes or unmutated specific methylated (hypo or hyper) genes or commonly methylated (hypo or hyper) genes between two prognostic groups (Fig. 2d and Additional file 2: Figure S1D). Notably, some important pathways were specifically deregulated in CLL such as ErbB, B cell receptor, PI3K-Akt, Wnt signaling, and MAP Kinase signaling (Fig. 2d and Additional file 2: Figure S1D). The detailed KEGG pathway summary with percentage of genes involved in each pathway along with the *p* values is listed in Additional file 3 (for B cell and PBMC comparisons) in the same order as in the corresponding heatmaps presented. Interestingly, most of these pathways like the B cell receptor, MAP Kinase, and PI3K-Akt pathways were also significant when analyzed for common cllDMGS from Additional file 2: Figure S2A between B cell and PBMC comparisons (Additional file 2: Figure S2C and Additional file 4).

### Correlation between candidate cllDMGs methylation and expression

**Fig. 3:**
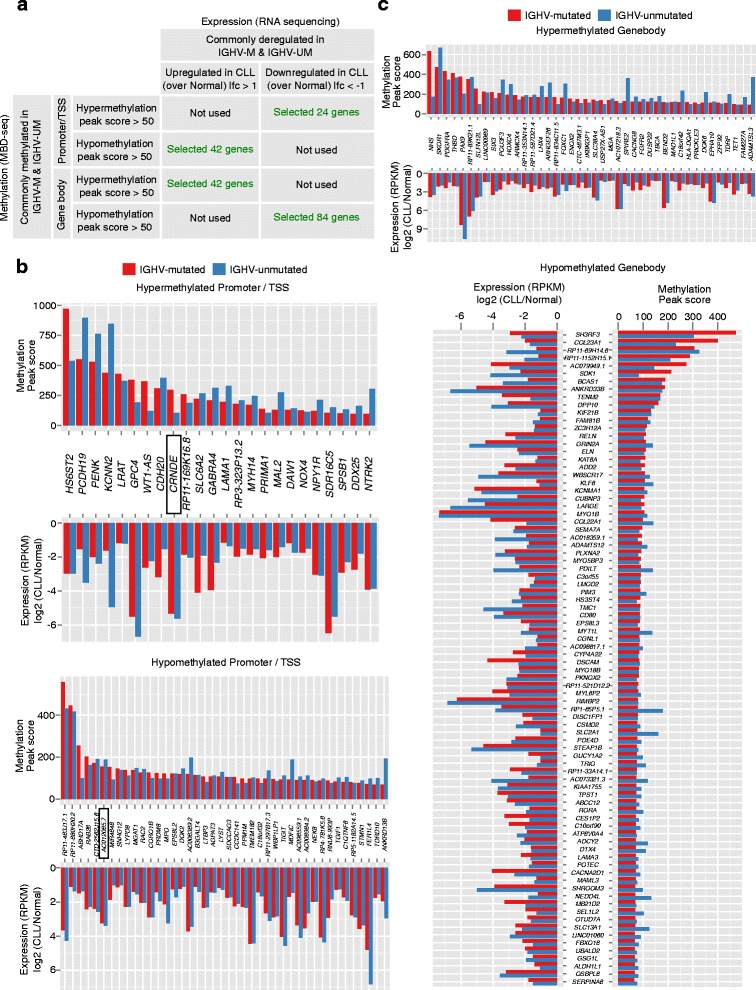
Regulation of cllDMGs by the distribution of methylation on gene structure and the gene expression patterns associated with methylation. **a** Table showing the selection of candidate genes depending on correlation between location of methylation on gene structure (promoter or gene body methylation in MBD-seq) and their pattern of gene expression (up or downregulated in RNA-seq, log2fold-change). The “selected number of genes” in *green* represents the candidate genes (cllDMGs) considered for further investigation. The two selected genes (*CRNDE* and *AC012065.7*) for further investigation from two categories were highlighted in bar graphs (**b** and **c**) with a *rectangle*. **b** The *top and bottom bar graphs* represent the list of selected cllDMGs from promoter-hypermethylated-downregulated and promoter-hypomethylated-upregulated patterns, respectively. **c** The *top and bottom bar graphs* show the list of selected cllDMGs from gene body-hypermethylated-upregulated and gene body-hypomethylated-downregulated patterns, respectively

Even though some of the cllDMGs were commonly hypermethylated or hypomethylated in all CLL patients, many genes showed significant differences in the methylation scores between the two prognostic groups. To further investigate the correlation between DNA methylation and gene expression, we used published RNA-seq data from a CLL cohort comprising 98 patients [23]. First, we selected a few commonly methylated cllDMGs in all CLL samples and sub-divided into two groups based on promoter methylation or gene body methylation [7] (Fig. 3a). There were nearly 24 cllDMGs that had promoter hypermethylation with lower gene expression and 42 cllDMGs with hypomethylated promoters showing higher gene expression. Consistent with recent analysis, gene body methylation positively correlated with gene expression [7]: 42 cllDMGs had hypermethylated gene body with higher gene expression and lower expression in the case of 84 cllDMGs with hypomethylated gene body. All the selected cllDMGs had greater than 50 peak score (in both IGHV-mutated and IGHV-unmutated groups) and with expression of log-fold change >±1 over normal B cell (in both IGHV-mutated and IGHV-unmutated groups) (Fig. 3b, c).

**Fig. 4:**
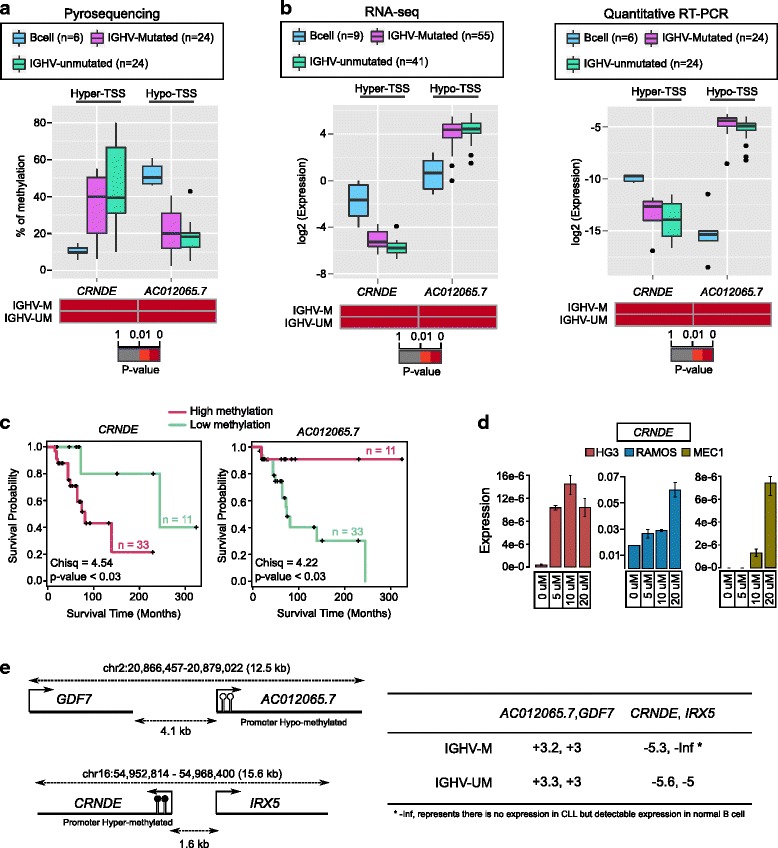
Validation of differential methylation and expression levels in CLL cohorts. **a** *Boxplots on top* shows the difference in distribution and level of methylation between IGHV-mutated, IGHV-unmutated, and sorted B cells for two selected genes (*CRNDE* and *AC012065.7*) obtained using pyrosequencing. **b** The *boxplots* shows the difference in gene expression levels between IGHV-mutated, IGHV-unmutated, and sorted B cells for same genes obtained using published RNA sequencing dataset (Ferreira PG et al.) and quantitative RT-PCR. The *heatmap below each boxplot* shows the significance level (*p* value) of the corresponding gene over B cell (IGHV-M, IGHV-mutated, and IGHV-UM, IGHV-unmutated). **c** Kaplan-Meier plots showing the clinical significance of all the validated genes based on high and low methylation levels. The high and low levels were calculated using upper and lower quartile based method for all the genes in total 44 CLL patient samples. **d** Gene expression levels of *CRNDE* using increasing concentrations of DAC treatment in different leukemic cell lines. **e** The illustrations (*left panel*) represents the protein coding genes *IRX5* and *GDF7* within 10-kb proximity of selected lncRNAs *CRNDE* and *AC012065.7*, respectively. The expression values for these lncRNAs and nearby protein coding genes are presented in *right panel* of the figure. The values in the panel represents log2-fold change in comparison between normal B cell and CLL groups (96 patients cohort, 55 IGHV-mutated, and 41 IGHV-unmutated), positive values means expression is more in CLL groups and vice versa

Since these selected genes from MBD-seq and RNA-seq data sets showed the expected correlation patterns between DNA methylation and gene expression, they serve as a vital resource for uncovering their possible role in CLL prognosis. In order to extend these observations, we selected two lncRNAs to further validate the significance of DNA methylation in gene expression using pyrosequencing and qRT-PCR methods respectively in an independent CLL cohort (the selected genes are highlighted in Fig. 3b). The selection of genes was based on both methylation peak scores and ncRNAs; however, we have excluded genes that were not suitable for designing pyrosequencing primers due to high CpG-rich regions. Since the significance of DNA methylation in the differential expression of lncRNAs has not been investigated in CLL, we selected lncRNAs are *CRNDE* and AC012065.7, which were hyper- and hypomethylated on promoter regions respectively (Fig. 3b). The methylation levels were validated using pyrosequencing in 48 CLL patients and 6 sorted B cell healthy controls, and as expected, we found that this data was in line with the MBD-seq data, where a higher percentage of methylation for all CLL samples was observed compared to normal samples (Fig. 4a). qRT-PCR analysis revealed that the DNA methylation levels of *CRNDE* and AC012065.7 showed an inverse correlation to gene expression levels in the same sample cohort (Fig. 4b, right panel), suggesting that these two cllDMRs may play a functional role in regulating the gene expression of cllDMGs. More importantly, our qRT-PCR data further corroborates with published independent RNA-seq data (with total 98 samples) (Fig. 4b, left panel).

We also analyzed the prognostic value of these two lncRNAs using Kaplan-Meier analysis. Both *CRNDE* and AC012065.7 lncRNAs showed a significant correlation between overall survival and DNA methylation in CLL patients (Fig. 4c). Higher methylation levels of the *CRNDE* promoter and lower methylation levels of the *AC012065.7* promoter correlated with poor overall survival (Fig. 4c).

Importantly, in order to explore the causal role of DNA hypermethylation in regulating *CRNDE* expression, we treated three different leukemic cell lines (HG3, MEC1, and RAMOS) with increasing concentrations of the methyl inhibitor (5′-Aza-2′-deoxycytidine, also known as DAC). As shown in Fig. 4d, a corresponding increase of *CRNDE* expression was demonstrated for all DAC treated samples compared to untreated samples in all the three cell lines, supporting that this gene is deregulated mainly due to hypermethylation on promoter region (Fig. 4d).

### LncRNA from cllDMGs show significant expression correlation with neighboring protein coding genes

We next investigated the expression correlation between lncRNAs from cllDMGs and nearby protein coding genes using RNA-seq datasets from 96 CLL patient cohorts. LncRNA *AC012065.7*, which is promoter hypomethylated with higher expression in CLL compared to normal (upregulated), showed positive expression correlation with nearby protein coding gene *GDF7* (Fig. 4e). *GDF7* is known to play an important role in growth, repair, and embryonic development, and its polymorphism leads to adenocarcinoma. Similarly, *CRNDE* also showed positive expression correlation with its neighboring protein coding gene *IRX5* (Fig. 4e). The gene *IRX5* has been shown to be involved in apoptosis and cell cycle regulation in prostate cancer cells [37]. Since the nearby protein coding genes of two selected lncRNAs has cancer related functions, we were interested in understanding the functional significance of nearby protein coding genes which are 10 kb proximity to all lncRNAs from cllDMGs (Additional file 7). The functional and cancer enrichment analysis revealed that cancer terms such as leukemia and lymphoma and KEGG pathways such as Wnt signaling (nearby genes from B cell and PBMC comparisons), pathways in cancer (B cell and PBMC comparisons), Hippo signaling (B cell and PBMC comparisons), transcription misregulation in cancer (B cell comparison), and NF-kappa B signaling (PBMC comparison) were significantly enriched (Additional file 7).

### Global methylation analysis of repetitive elements in the IGHV-mutated and IGHV-unmutated CLL samples

**Fig. 5:**
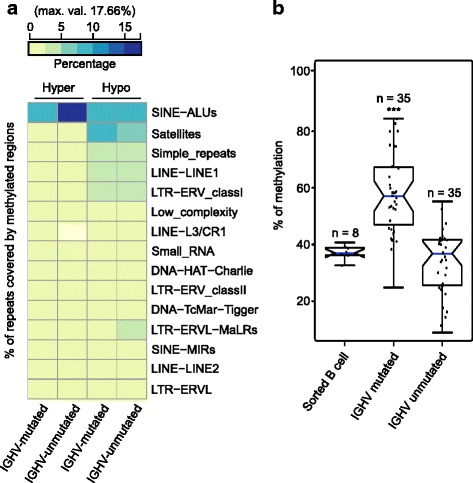
CLL-associated differentially methylated repeat elements (DMrE) over normal sorted B cell. **a** The heatplot represents the enrichment of cllDMRs over different repeat elements in B cell comparison. **b** Validation of IGHV-mutated specific hypermethylated SINE-ALU repeat region in 70 CLL patient samples and 8 normal B cell controls using pyrosequencing method. Statistical significance was derived using unpaired Student’s *t* test, *p < 0.05, **p < 0.01, and ***p < 0.001

Global hypomethylation in cancer cells can be largely attributed to reduced methylation of repetitive elements in the genome [38–40]. To this end, we investigated the percentage of repeat sequence covered by the cllDMRs and found that SINE-ALUs, satellites, simple repeats, and LINEs which were enriched in significantly hypomethylated regions from CLL samples compared to the normal controls (Fig. 5a). Moreover, the IGHV-unmutated poor prognostic CLL samples showed less enrichment of these repeat elements in hypomethylated regions compared to the IGHV-mutated CLL samples, further supporting the hypothesis that SINE and satellite repeats are hypermethylated in healthy normal controls and IGHV-mutated samples. We also investigated if repetitive elements were enriched in hypermethylated regions of CLL samples compared to normal and found enrichment of SINE-ALUs in all CLL samples. Interestingly, these SINE-ALUs were more in hypermethylated regions from the IGHV-unmutated group compared to the IGHV-mutated group (Fig. 5a and Additional file 2: Figure S3A). We selected one methylated SINE-ALU repeat sequence which was more enriched in the IGHV-mutated prognostic group compared to the IGHV-unmutated prognostic group in our analysis and validated it using pyrosequencing (Fig. 5b).

## Discussion

Using a high-throughput affinity-based methylated DNA-enrichment technique, for the first time, we analyzed global methylomes of two different CLL prognostic groups to identify DNA methylation based protein coding, lncRNA, and repeat RNA signatures by comparing to two different kinds of healthy normal controls; both sorted B cells and PMBCs. More than half of the cllDMGs were revealed to be common between B cell and PMBC comparisons, sharing many significant common biological pathways. These observations suggest that the common differentially methylated genes from these two comparisons could be a huge resource for investigating epigenetic-based signatures for CLL pathogenesis. According to recent publications based on 450K methylation array data, CLL has been stratified into three groups with similarity to naïve B cells, memory B cells, and an intermediate group [7, 41]. However it is also true that many known prognostic markers and candidate genes were identified in CLL by comparing with normal sorted B cells, such as ZAP70 [42], BCL2 [43], and ANGPT2 [36]. Moreover, the exact corresponding healthy control for CLL is not clear as the cell of origin of this B cell leukemia is still under debate. Therefore, based on many recent published global methylation studies in CLL [3, 5, 8, 44] where they used normal B cell as controls, we also used sorted B cells and PBMCs in our study to identify CLL-associated hyper/hypo methylated genes.

Several lines of evidence suggest that CLL genomes are hypomethylated compared to normal sorted B cells [45–48]. Since MBD-Seq investigates methylation on the genome-scale in an unbiased manner, it is an ideal methodology to address the global CpG methylation levels in CLL subsets in relation to sorted B cells and PBMCs. We found that IGHV-unmutated samples exhibit significant overall global hypomethylation compared to IGHV-mutated samples, whose methylation levels were comparable to normal healthy controls, which is consistent with their favorable clinical prognosis. Interestingly, when we compared the distribution of cllDMRs across the genome, we observed that hypermethylated cllDMRs were enriched in promoter regions, whereas hypomethylated cllDMRs were significantly enriched over gene body and intergenic regions further supporting the above statement.

Moreover, this is the first detailed study where both CLL-associated hypermethylated and hypomethylated cllDMRs were investigated in unbiased manner across genic, intergenic and repeat regions of the genome. Unlike MeDIP-seq, which enriches regions with relatively lower CpG density, MBD-seq mostly enriches regions with slightly higher CpG density [49]. Interestingly, all the cllDMRs showed high GC content (more than 50 to 55 %), which was expected based on above mentioned study (the percentage of GC content and CpG content for all the cllDMRs are mentioned in the Additional files 5 and 6 for both B cell and PBMC comparisons, respectively). In this study, we also investigated the grade of enrichment of the cllDMGs in other cancer types and found that CLL was the top-listed among the leukemias (Fig. 2c). Also, the enrichment of common cllDMGs in other lymphomas and cancers such as non-Hodgkin lymphoma, colorectal, and prostate cancer indicates that these could be signature DMRs for cancers in general, including CLL.

Lately, there has been a clear shift in researchers’ focus towards lncRNAs and understanding their role in cancer initiation and progression [50, 51]. However, their relative importance in hematological disorders is still limited. A recent RNA-seq based CLL study identified many differentially expressed lncRNAs as potential biomarkers in CLL pathogenesis [23]; however, the mechanisms underlying their differential expression is still unknown. In the current study, a significant portion (nearly 40 % of hypermethylated and 60 % of hypomethylated) of differentially methylated transcripts were ncRNA, comprising small ncRNAs like microRNAs, snRNAs, snoRNAs and lncRNAs, such as lncRNAs, pseudogenes and antisense transcripts. This large dataset of ncRNAs could also be a resource for further studies, aiming at understanding the functional role of ncRNA in CLL pathogenesis. Towards this end, we validated the differential expression of two lncRNAs (*AC012065.7*and *CRNDE*) in an independent cohort. Both these lncRNAs were found to be hyper and hypomethylated using both normal B cell and PBMC comparisons as listed in Additional file 6.

Another important aspect of the current study, unlike previously published methylation array studies, is that we have performed extensive correlation studies of cllDMG methylation and gene expression using the published RNA-seq data set from 98 CLL patients [23]. We found several protein coding RNAs and lncRNAs showing strong correlation between DNA methylation and gene expression. For example, cllDMGs with hypermethylation of the promoter had lower gene expression levels, whereas cllDMGs with gene body hypomethylation had higher gene expression (Fig. 3). Our qRT-PCR and pyrosequencing has validated the gene expression and DNA methylation levels, respectively, of selected lncRNAs (*CRNDE* and *AC012065.7*). Moreover, hypermethylation and hypomethylation of *CRNDE* and *AC012065.7* lncRNAs, respectively, correlated with inferior overall survival, and since there are no other lncRNAs identified in CLL as epigenetic prognostic markers, it would be interesting to further investigate these two lncRNAs for their potential prognostic role in CLL.

Finally, we found that several KEGG pathways in CLL, including MAPK, PI3K-AKt, and B cell receptor signaling, were enriched with cllDMGs. Moreover, the analyses of CLL samples in relation to both B cell and PBMC normal controls revealed several common KEGG pathways, and many of these pathways were also listed in a recent RNA-seq study in CLL [23], implying that methylation could be a determining factor in the aberrant regulation of these pathways. Also, some of these pathways, like Notch [48, 52, 53] and NF-kappa B [53, 54] have already been implicated in CLL.

Transposable elements such as LINEs and SINEs are enriched with CpG sites and therefore DNA methylation levels of these repeat regions serve as a robust surrogate marker of global DNA methylation [38, 40]. CpG methylation analysis of repeat sequences is not possible with bisulfite converted microarray-based techniques. Hence, until now, data about the possible relevance of repeat region methylation in CLL has been scarce. We found that many repeat regions like SINE/Alus, LINE/Alus, LTR/ERV, satellites, and simple repeats were significantly hypomethylated in both prognostic CLL subgroups (Fig. 5a). Considering that LINE repeats along with SINE/Alu repeats constitute more than 40 % of the total human genome, it is generalized that hypomethylation of these repeats results in global demethylation [55]. On the other hand, we also identified specific SINE/Alu repeats which were significantly hypermethylated in both IGHV-mutated and IGHV-unmutated CLL subgroups against normal B cell controls, indicating a pathogenic function of hypo/hypermethylated specific SINE/Alu repeats in CLL. The exact mechanism by which these repeat regions may increase the risk of cancer is unclear; however, it has been hypothesized that cells with higher methylation levels may have a longer survival, and thus, combined with carcinogen exposure, this methylation pattern may favor clonal expansion of damaged cells [56]. We then validated one of the IGHV-mutated specific hypermethylated SINE/Alu repeat region using pyrosequencing in a larger CLL cohort comprising 70 tumor samples. However, we could not validate SINE/Alu repeat regions that were more hypermethylated in IGHV-unmutated samples due to a high GC content. So, further work is needed to realize their significance as prognostic biomarkers in CLL.

## Conclusions

In summary, for the first time using MBD-Seq, we investigated global CLL-specific methylomes using sorted B cells and PBMCs as controls. We identified several lncRNAs, including *CRNDE* and *AC012065.7*, repetitive elements (SINE/Alu), and protein coding RNAs harboring cllDMRs with a potential role in CLL disease pathogenesis and/or prognosis. Also, our data opens up several important CLL-associated pathways for further investigations.

## Additional files

Additional file 1: Additonal data 1. (XLS 31.5 kb)
Additional file 2: .Additonal figures and information file. (PDF 731 kb)
Additional file 3: Additonal data 2. (XLS 172 kb)
Additional file 4: Additonal data 3. (XLS 29.5 kb)
Additional file 5: Additonal data 4. (XLS 3951 kb)
Additional file 6: Additonal data 5. (XLS 5827 kb)
Additional file 7: Additonal data 6. (XLS 231 kb)

## Abbreviations

CLL: Chronic lymphocytic leukemia
CpG: C (cytosine) base followed immediately by a G (guanine) base
DMGs: Differentially methylated genes
DMRs: Differentially methylated regions
IGHV: Immunoglobulin heavy chain variable region genes
LncRNA: Long noncoding RNA
MBD-seq: Methyl-CpG-binding domain protein enriched genome-wide sequencing
PBMC: Peripheral blood mononuclear cells
