# Supplementary material for: Global DNA methylation profiling reveals new insights into epigenetically deregulated protein coding and long noncoding RNAs in CLL
Source: Clin Epigenetics. 2016 Oct 12;8:106. doi: 10.1186/s13148-016-0274-6 (PMC5062931; doi:10.1186/s13148-016-0274-6)
Supplement: Additional file 2: — .Additonal figures and information file. (PDF 731 kb) [file 13148_2016_274_MOESM2_ESM.pdf]

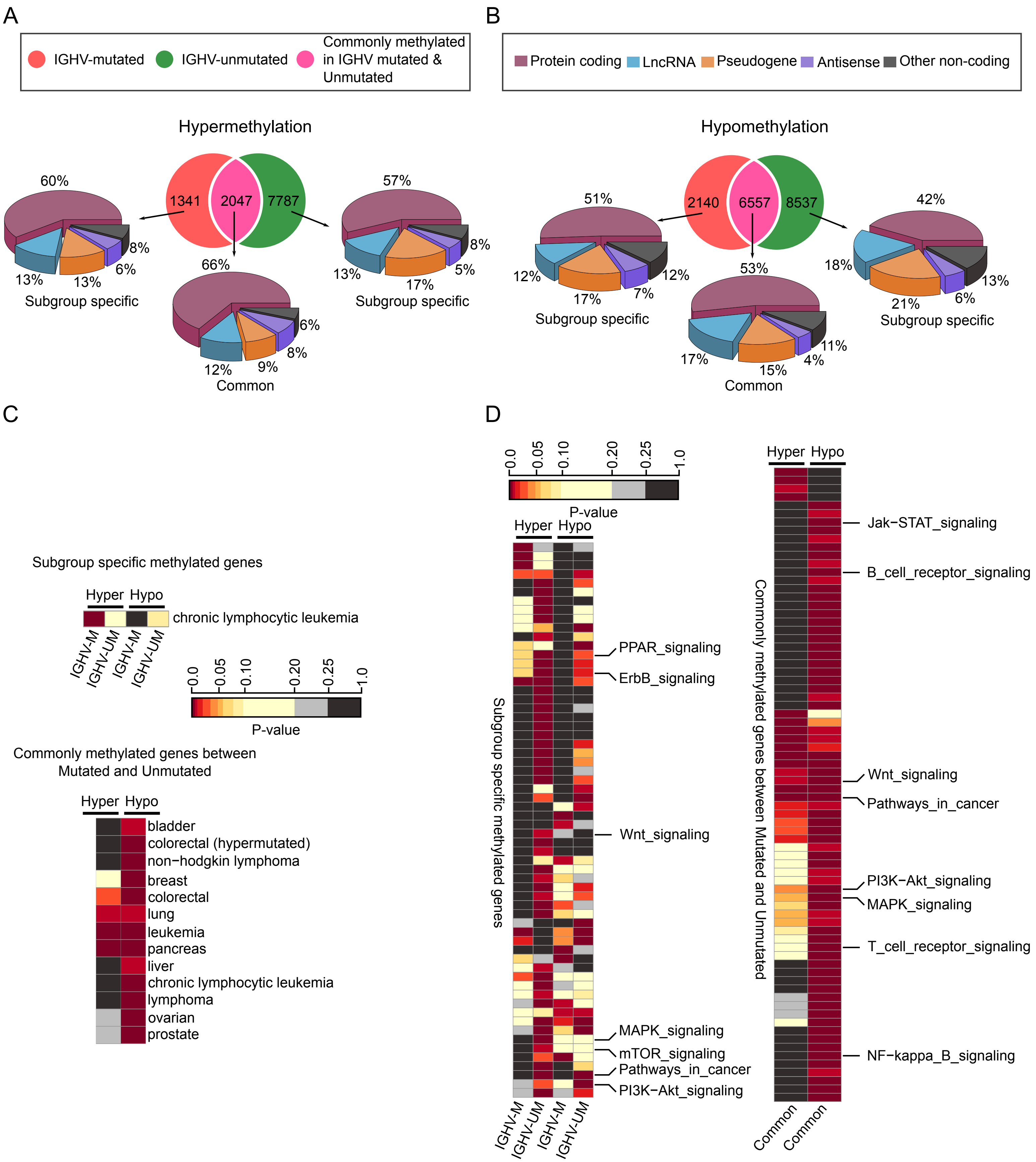
A
IGHV-mutated
IGHV-unmutated
Commonly methylated in IGHV mutated & Unmutated
Hypermethylation
1341
2047
7787
60%
8%
6%
13%
13%
Subgroup specific
57%
8%
5%
13%
17%
Subgroup specific
66%
6%
8%
12%
9%
Common
B
Protein coding
LncRNA
Pseudogene
Antisense
Other non-coding
Hypomethylation
2140
6557
8537
51%
12%
12%
17%
7%
Subgroup specific
42%
18%
13%
21%
6%
Subgroup specific
53%
11%
17%
4%
15%
Common
C
Subgroup specific methylated genes
Hyper
Hypo
chronic lymphocytic leukemia
IGHV-M
IGHV-UM
IGHV-M
IGHV-UM
0.0
0.05
0.10
0.20
0.25
1.0
P-value
Commonly methylated genes between Mutated and Unmutated
Hyper
Hypo
bladder
colorectal (hypermutated)
non−hodgkin lymphoma
breast
colorectal
lung
leukemia
pancreas
liver
chronic lymphocytic leukemia
lymphoma
ovarian
prostate
D
0.0
0.05
0.10
0.20
0.25
1.0
P-value
Hyper
Hypo
Subgroup specific methylated genes
PPAR_signaling
ErbB_signaling
Wnt_signaling
MAPK_signaling
mTOR_signaling
Pathways_in_cancer
PI3K−Akt_signaling
IGHV-M
IGHV-UM
IGHV-M
IGHV-UM
Hyper
Hypo
Commonly methylated genes between Mutated and Unmutated
Jak−STAT_signaling
B_cell_receptor_signaling
Wnt_signaling
Pathways_in_cancer
PI3K−Akt_signaling
MAPK_signaling
T_cell_receptor_signaling
NF−kappa_B_signaling
Common
Common

Supplementary Figure 1. Association of DMRs to genes and importance of associated genes (differentially methylated genes, DMGs) in CLL over normal PBMC. A,B) Venn diagram shows the overlap of differentially methylated genes (DMGs, hypermethylated and hypomethylated) between IGHV mutated and unmutated groups. And the pie chart represents the percentage of different classes of genes such as protein coding, lncRNA, pseudogenes, antisense and other noncoding RNAs. **C**) The heatmap shows enrichment of cllDMGs (top; subgroup specific and bottom; common DMGs) in different cancer types from Network of Cancer Genes (NCG 4.0). The cancer types are assigned and ranked using GeneSCF. The presented enrichment was filtered using p-value < 0.01 with at-least 5 % of total cancer genes covered by DMGs. **D**) The heatmap shows the KEGG pathways obtained using cllDMGs from IGHV mutated and IGHV unmutated prognostic groups. The pathways were assigned and ranked using GeneSCF. The presented pathways are filtered using p-value < 0.01 with at-least 5 % of total pathway genes covered by DMGs (see methods). The left side of the heatmap represents the subgroup specific (IGHV mutated and IGHV unmutated) hyper and hypomethylated associated pathways; and the right side of the heatmap for common DMGs between IGHV mutated and IGHV unmutated groups (see methods).

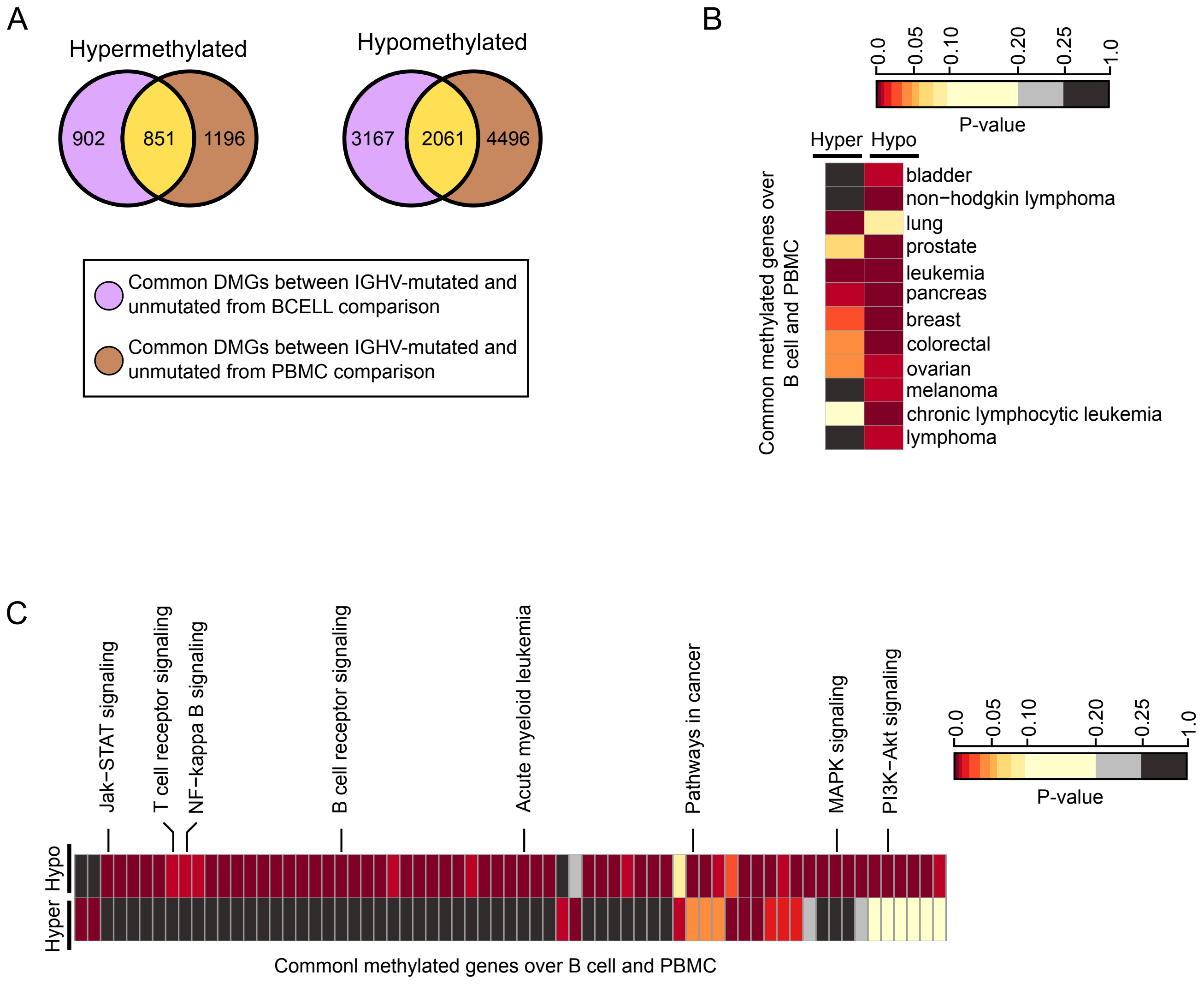

Supplementary Figure 2. Significance of common cllDMGs between B cell and PBMC comparisons. A) The venn diagram shows the overlap of common differentially methylated genes (IGHV mutated and IGHV unmutated prognostic groups) between B cell and PBMC comparisons. The left panel of venn diagram shows the overlap for hypermethylated genes and the right panel for hypomethylated genes. **B**) The heatmap shows enrichment of common cllDMGs between B cell and PBMC comparisons in different cancer types from Network of Cancer Genes (NCG 4.0). The cancer types are assigned and ranked using GeneSCF. The presented enrichment was filtered using p-value < 0.01 with at-least 5 % of total cancer genes covered by cllDMGs. **C**) The heatmap shows the KEGG pathways obtained using common cllDMGs between B cell and PBMC comparisons. The pathways were assigned and ranked using GeneSCF. The presented pathways are filtered using p-value < 0.01 with at-least 5 % of total pathway genes covered by DMGs (see methods).

A

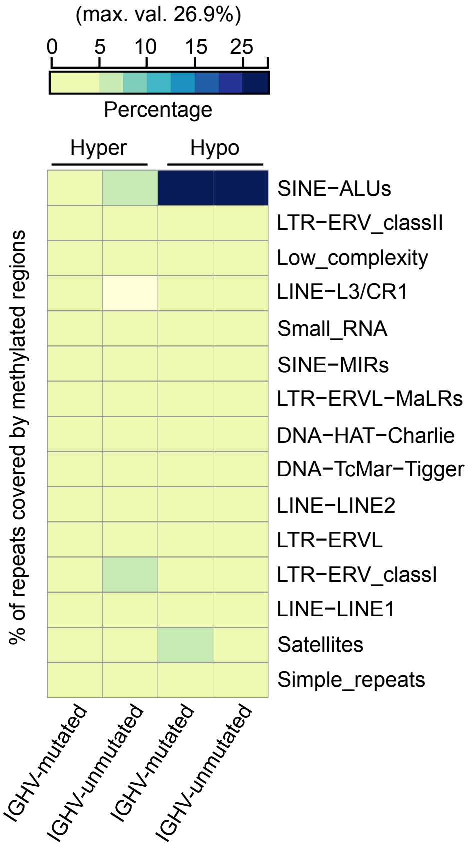

Supplementary Figure 3. Associated differentially methylated repeat elements (DMrE) over normal PBMC. A) The heatplot represents the enrichment of clDMRs over different repeat elements in PBMC comparison.

SUPPLEMENTARY DATASETS

Supplementary data 1A: IGHV mutated specific CLL hypermethylated genes in B cell comparison

Supplementary data 1B: IGHV unmutated specific CLL hypermethylated genes in B cell comparison

Supplementary data 1C: Commonly hypermethylated genes between IGHV mutated and unmutated prognostic groups in B cell comparison

Supplementary data 1D: IGHV mutated specific CLL hypomethylated genes in B cell comparison

Supplementary data 1E: IGHV unmutated specific CLL hypomethylated genes in B cell comparison

Supplementary data 1F: Commonly hypomethylated genes between IGHV mutated and unmutated prognostic groups in B cell comparison

Supplementary data 1G: Summary of obtained reads from CLL groups and normal sample groups.

Supplementary data 2A: IGHV mutated specific CLL hypermethylated genes in PBMC comparison

Supplementary data 2B: IGHV unmutated specific CLL hypermethylated genes in PBMC comparison

Supplementary data 2C: Commonly hypermethylated genes between IGHV mutated and unmutated prognostic groups in PBMC comparison

Supplementary data 2D: IGHV mutated specific CLL hypomethylated genes in PBMC comparison

Supplementary data 2E: IGHV unmutated specific CLL hypomethylated genes in PBMC comparison

Supplementary data 2F: Commonly hypomethylated genes between IGHV mutated and unmutated prognostic groups in PBMC comparison

Supplementary data 3A: Cancer type enrichments of subgroup specific (IGHV mutated and IGHV unmutated) cllDMGs from B cell comparison.

Supplementary data 3B: Cancer type enrichments of common cllDMGs between IGHV mutated and IGHV unmutated prognostic groups from B cell comparison.

Supplementary data 3C: Cancer type enrichments of subgroup specific (IGHV mutated and IGHV unmutated) cllDMGs from PBMC comparison.

Supplementary data 3D: Cancer type enrichments of common cllDMGs between IGHV mutated and IGHV unmutated prognostic groups from PBMC comparison.

Supplementary data 3E: KEGG pathway enrichments of subgroup specific (IGHV mutated and IGHV unmutated) cllDMGs from B cell comparison.

Supplementary data 3F: KEGG pathway enrichments of common cllDMGs between IGHV mutated and IGHV unmutated prognostic groups from B cell comparison.

Supplementary data 3G: KEGG pathway enrichments of subgroup specific (IGHV mutated and IGHV unmutated) cllDMGs from PBMC comparison.

Supplementary data 3H: KEGG pathway enrichments of common cllDMGs between IGHV mutated and IGHV unmutated prognostic groups from PBMC comparison.

Supplementary data 4A: KEGG pathway enrichments of common cllDMGs between B cell and PBMC comparisons.

Supplementary data 4B: Cancer type enrichments of common cllDMGs between B cell and PBMC comparisons.

Supplementary data 5A: The list of nearby protein coding genes within 10 kb proximity to lncRNAs from cllDMGs from B cell comparison.

Supplementary data 5B: KEGG pathway enrichments of nearby protein coding genes to lncRNAs from cllDMGs in B cell comparisons.

Supplementary data 5C: Cancer enrichments (NCG) of nearby protein coding genes to lncRNAs from cllDMGs in B cell comparisons.

Supplementary data 5D: The list of nearby protein coding genes within 10 kb proximity to lncRNAs

from cllDMGs from PBMC comparison.

Supplementary data 5E: KEGG pathway enrichments of nearby protein coding genes to lncRNAs from cllDMGs in PBMC comparisons.

Supplementary data 5F: Cancer enrichments (NCG) of nearby protein coding genes to lncRNAs from cllDMGs in PBMC comparisons.

Supplementary data 6A: Information on CLL patient samples used for MBD seq and validation experiments.

Supplementary data 6B: Clinical data for the CLL patient samples used for survival analysis**.**
